# Genetics education in primary care residency training: satisfaction and current barriers

**DOI:** 10.1186/s12875-022-01765-0

**Published:** 2022-06-19

**Authors:** Nadia Falah, Amna Umer, Emilea Warnick, Manuel Vallejo, Timothy Lefeber

**Affiliations:** 1 Department of Pediatrics, Division of Genetics, 1 Medical Center Drive, West Virginia Medicine Children’s Hospital, West Virginia University School of Medicine, Morgantown, WV 26506 USA; 2 West Virginia University Cancer Institute, Morgantown, WV 26506 USA; 3 Department of Pediatrics, West Virginia University Robert C. Byrd Health Sciences Center, Morgantown, WV 26506 USA; 4 Graduate Medical Education, West Virginia School of Medicine, Morgantown, WV 26506 USA

**Keywords:** Genetics education, Rare Disease, Residency, Primary Care, Precision Medicine, Personalized Medicine

## Abstract

**Background:**

Genetics education can be integrated into general care medicine through primary care residency programs. A study of primary care residents was done to evaluate quality, satisfaction, and barriers in genetics education in residency training programs. Thus, providing more evidence for the necessity for its development and progress.

**Methods:**

A cross-sectional descriptive self-administered questionnaire survey was delivered to four primary care West Virginia University (WVU) residency training programs in 2020–2021. The anonymous 14-item survey included the following questionnaire domains: general data, genetics training satisfaction, and genetics education barriers.

**Results:**

The survey response rate was 52% (70/123) and 59 participants completed the survey. Overall, respondents viewed genetic education as critical to their chosen specialty (90%). Trainees at all educational levels obtained their education mostly from class based educational curricula (77% from lectures, 65% from didactic and 49% from grand rounds). The majority of survey respondents indicated insufficient experience with genetic patient care (34% ward genetic consultation, 5% clinic experience, 0% genetic department rotation). The percentage of residents who were satisfied with genetic topics were as follows: basic genetics (57%), capturing family history (82%), initiating basic genetic workup (15%), a basic understanding of the genetic report (23%), basic management surveillance in the genetic patient (18%), understanding the genetic referral and explaining it to a patient (47%).

Residents reported barriers to genetic interest included complexity of the field (87%), followed by limited utility of genetics testing (41%). The most common suggestions for improving the genetic education component were to provide more lectures (61%), followed by enhanced advertisement of genetic education resources specifically rotations in the genetics department (22%). Other suggestions include the integration of genetic education in inpatient learning (20%) and providing research experience (7%).

**Conclusion:**

Primary care residents were satisfied with their genetic knowledge in the classroom and stated a clear need for enhanced hands-on clinical skills and research experience in their current residency training. The survey suggestions for improvement can enhance primary care residents’ genetic training that can lead to advances in rare disease recognition, precision medicine, and improve access to genetics testing.

**Supplementary Information:**

The online version contains supplementary material available at 10.1186/s12875-022-01765-0.

## Introduction

**Fig. 1:**
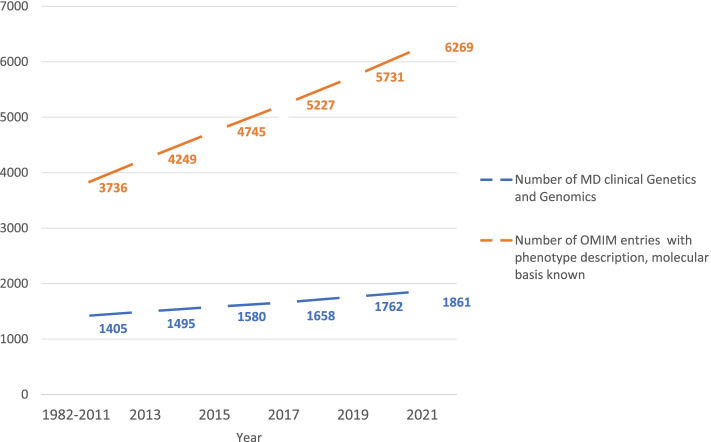
The change in the number of MD board certified geneticist over the years as reported by American Board of Medical Genetics and Genomics (ABMGG) to the number of increases in the rate of genetic discovery as reported by the Online Mendelian Inheritance in Man (OMIM) database

As the science of genetics expands, the demand for genetic specialists has outpaced the availability of genetic professionals (Fig. 1). A recent study examining the genetic workforce in the United State (USA) concluded a gap between clinical geneticist and the genetics service needed to increase patient demand [1]. Therefore, when access to a geneticist is not a possibility, there is increased focus placed on Primary Care Physicians (PCP), who can be involved in genetic service referrals as well as genetic testing [2]. Despite the advance in the genetic testing, it is known that conventional genetic education in primary care has not changed over the previous decades [3–5]. It is therefore recommended that new training changes be made in primary care residency programs [6].

The American Association of Family Practice (AAFP) define a Primary Care Physician (PCP) as a physician who provides definitive care to the undifferentiated patient at the point of first contact and takes continuing responsibility for providing the patient's comprehensive care [7]. These physicians are specifically trained to provide comprehensive primary care services through residency or fellowship in family medicine, general internal medicine, or general pediatrics [7]. Residency training programs require rotations in multiple subspecialities and offer an elective rotation that allow trainees to get more experience in the subspecialities of their interest. Genetics rotation is part of an elective rotation [8, 9]. In addition, residents are exposed to genetic education through ward consultation, classroom lectures, and research that includes topics pertinent to genetics.

Genetic testing and service are no longer restricted to rare diseases; they are increasingly essential in the diagnosis and management of medical disorders such as congenital malformations, developmental delay evaluations, cancer, prenatal care, and neurological problems [10, 11]. There is growing evidence that primary care doctors would benefit from better understanding of the options for early disease detection, availability of genetics testing, results interpretation, and prevention, management, and treatment strategies used by practitioners from a variety of disciplines [12].

Recent research has revealed significant challenges in offering genetic education in medicine [13]. PCPs lack the knowledge and skills needed to properly administer genetic services, and these practitioners offer genetic testing only if it would benefit their patients [13]. Furthermore, these providers observed a lack of knowledge and awareness of genetics [13, 14]. The American College of Medical Genetics (ACMG) has created a 101 genetic education online continuing medical education (CME) course for nongenetic health care practitioners in the goal of closing the gap and encouraging the integration of genetics services into primary care [15].

Genetics education may be integrated into general care medicine through primary care residency programs. Yet our understanding of satisfactions and barriers of current genetics education in primary-care training has not been elucidated.

## Objectives

The purpose of this study was to assess residents' genetics education, satisfaction, as part of their residency training programs, as well as to identify potential barriers, and make recommendations for future paths in enhancing genetics education.

## Study design and methods

A self-administered anonymous questionnaire with 14 survey items was issued to four primary care residency program trainees (2020–2021) from WVU Medicine in Morgantown, West Virginia: Pediatrics, Internal Medicine, Medicine-Pediatrics, and Family Medicine. Program directors and the office of medical education circulated the electronic survey through email. A multidisciplinary team that included a geneticist, a pediatrician, and a graduate medical education leader created the survey. The questions covered the following topics: demographic information (six questions), genetics interest and experience (five questions), training satisfaction (one question with five-point Likert scales including five satisfaction topics), and genetics education barriers (one multiple-choice question with open-ended questions) and recommendations (one question). Supplement 1 contains the survey questions. How were the survey questions agreed upon?

Data from completed questionnaires were gathered and analyzed using Qualtrics and Microsoft Excel. The five-point Likert scales were condensed into binary data by combining: "satisfied /very satisfied" for those who were satisfied with their genetics education and "not satisfied /very unsatisfied" for those who were dissatisfied. The survey was recirculated four times with at least two weeks interval. The first 50 participants received a $10 gift card as appreciation, once a completed questionnaire was received. This study was approved by WVU Institutional Review Board Committee (Protocol number 2103261670).

## Results

### General data

In total, 70 residents responded to the survey (52% response rate, *n* = 135) (Table 1.) in four primary care residency training programs. Only 59 residents completed the survey; 22 Internal Medicines, 14 Pediatrics, 11 Medicine and Pediatrics, and 12 Family Medicine. Demographic information is presented in Table 1.

**Table 1:** Demographics of the participants

	Family Medicine	Internal Medicine	Med-Peds Resident	Pediatrics Resident
PGY				
PGY1	4	9	5	8
PGY2	5	9	3	2
PGY3	3	4	2	4
PGY4	0	0	1	0
Age				
25–30	6	16	11	12
31–35	4	2	0	2
36–40	1	1	0	0
41–45	1	2	0	0
46–50	0	1	0	0
Gender				
Female	7	12	8	12
Male	5	10	3	2
Ethnicity				
Asian	0	6	0	2
Black/African	0	1	0	0
Middle Eastern	0	1	0	0
White	12	14	11	11
Medical school				
International	1	3	0	1
USA	11	19	11	12

### Genetics education and experience

**Fig. 2:**
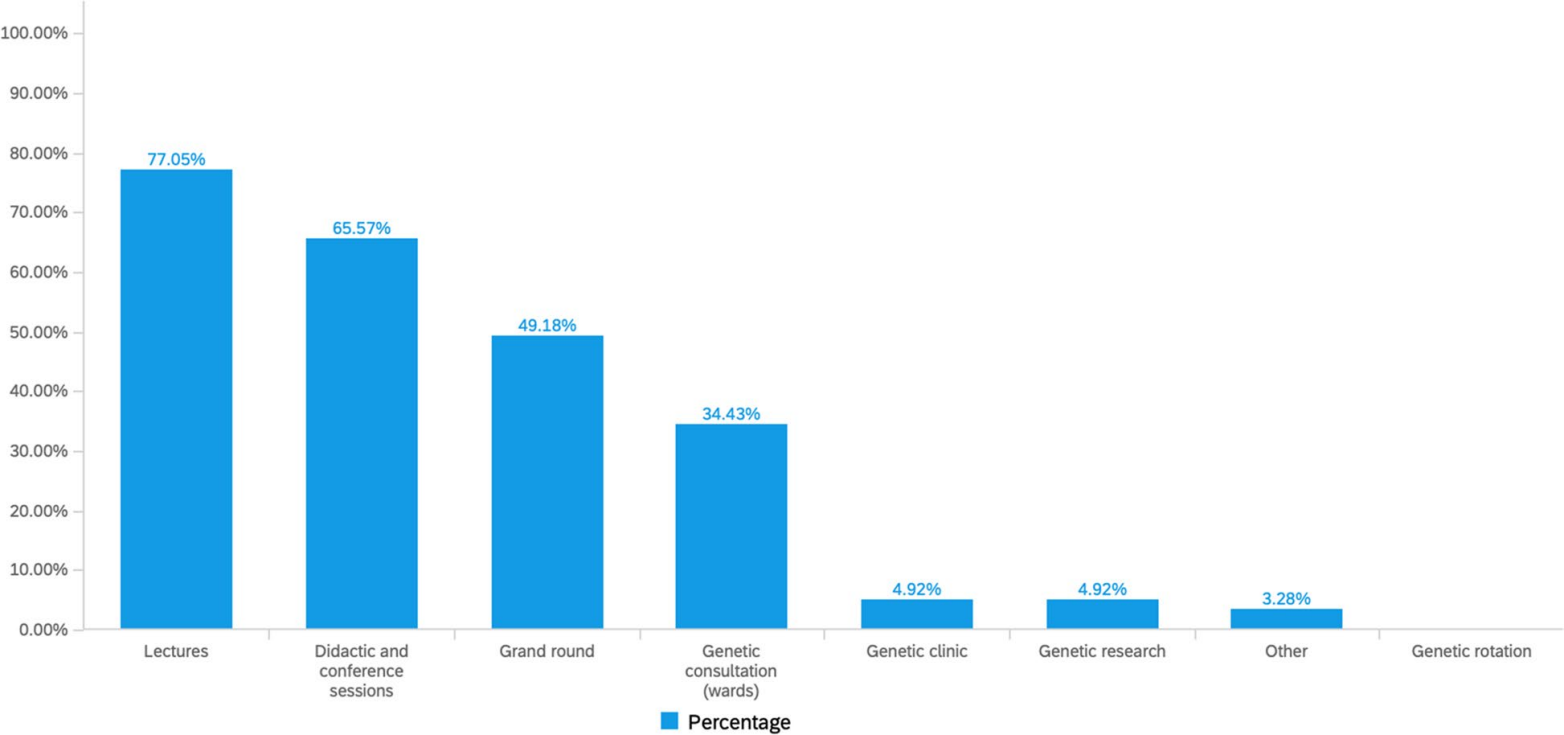
Genetics education experience as reported by all residents in primary care residency training at WVU Medicine

**Fig. 3:**
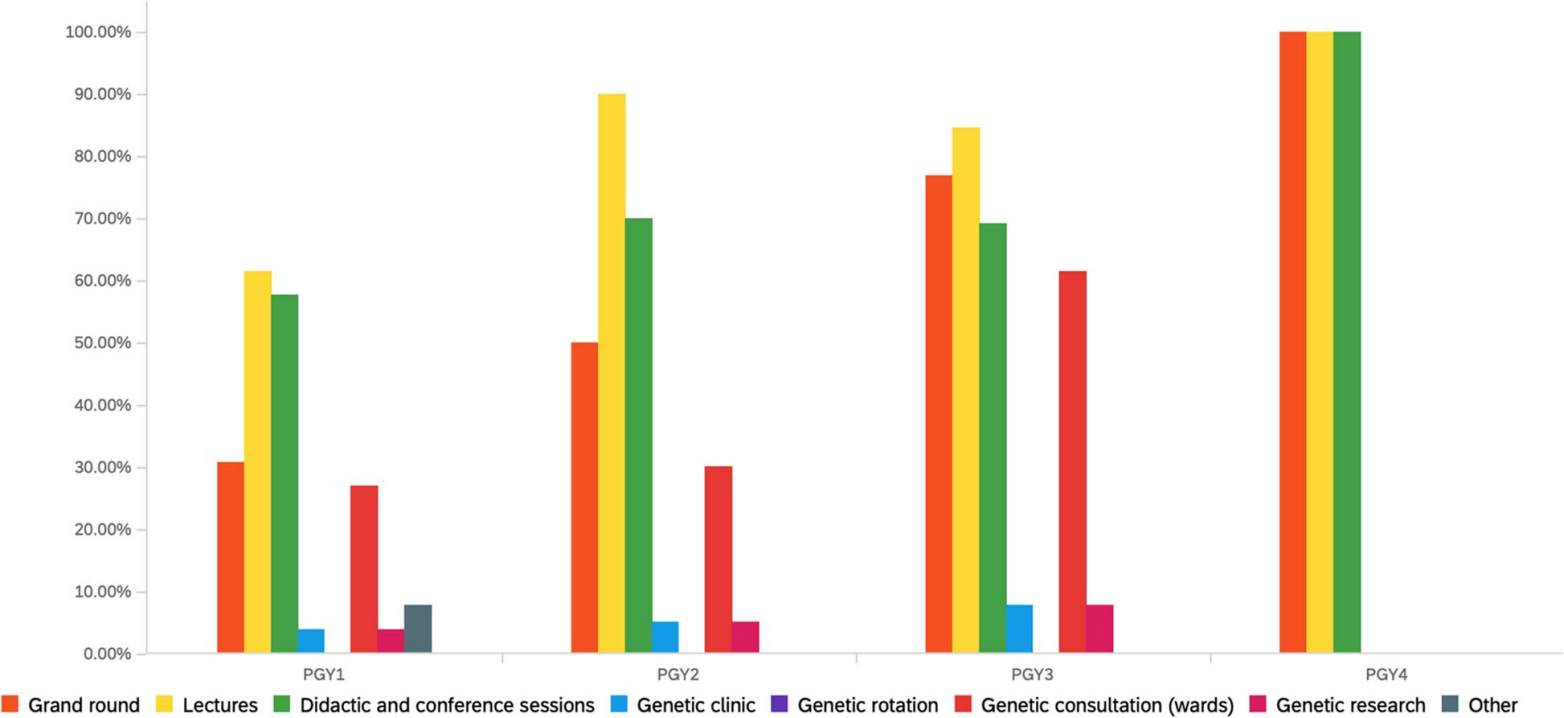
Genetics education experience as reported by PGY level in primary care residency training at WVU Medicine

Overall, the respondents described genetic education as essential to any primary care chosen specialty (90%). The majority received their genetic experience through a classroom-based education (77% lectures followed by 66% didactics, 49% grand rounds). The majority received no or limited hands-on clinical experience, 34% had a genetic inpatient ward experience, 5% has a genetic clinic experience, none of the respondents had completed a genetic rotation (Fig. 2–3).

### Genetics training satisfaction

**Fig. 4:**
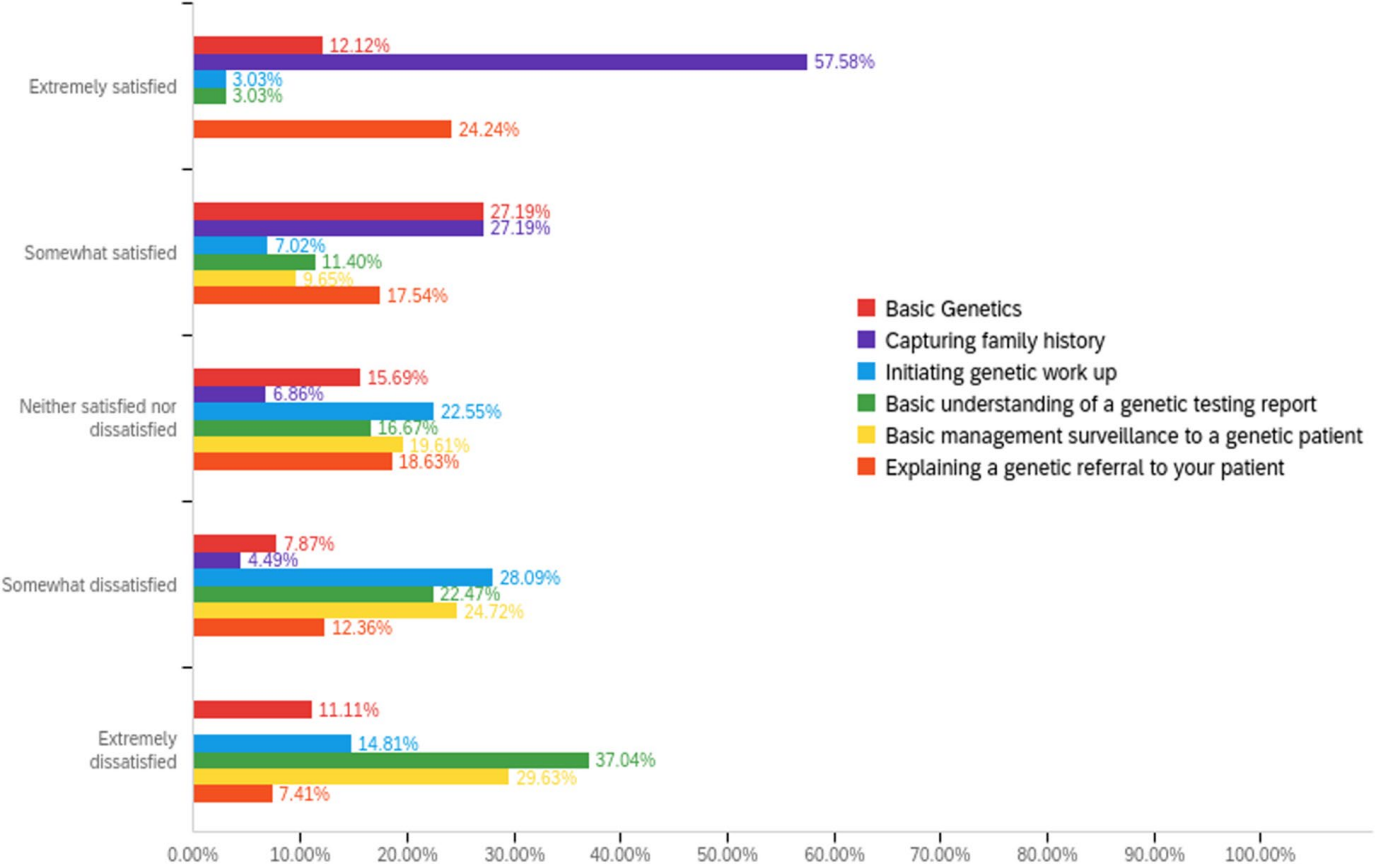
Genetics education satisfaction as reported by all participant residents in primary care residency training at WVU Medicine

The following is the percent of residents who were satisfied in the surveyed genetic domains: basic genetics (57%), capturing family history (82%), initiating basic genetic workup (15%), a basic understanding of the genetic report (23%), basic management surveillance in the genetic patient (18%), understand the genetic referral and explaining it to a patient (47%). Figure 4, Table 2.

**Table 2:** Genetics education satisfaction as reported by all participant residents in primary care residency training that includes the neutral response

Field	Satisfied	Neither satisfied nor satisfied	dissatisfied
Basic Genetics	57%	26.23%	16%
Capturing family history	82%	11.48%	7%
Initiating genetic work up	15%	37.70%	48%
Basic understanding of a genetic testing report	23%	27.87%	49%
Basic management surveillance to a genetic patient	18%	32.79%	49%
Explaining a genetic referral to your patient	47%	31.67%	22%

### Genetic education barriers and suggestions for improvement

Residents reported the complexity of the field as a barrier to genetic education (87%), followed by the limited utility of genetic testing (41%), and 11% found genetics of rare disease, not an area of interest. More lectures (36 residents, 61%) were suggested as a way to improve the genetic education component, while 13 residents (22%) suggested rotation in the genetic division (2 residents suggested short a rotation, and 8 residents suggested increased awareness of rotations). There were 12 residents (20%) who proposed incorporating genetic education into inpatient learning. Four residents (7%) proposed learning genetics through research experience.

## Discussion

Assessing genetic education as part of a primary care training program allows for the identification of knowledge gaps and the implementation of training-level improvement strategies. Although trainees believed that genetics was important for their future practice, there were significant gaps in hands-on clinical experience during their clinical training. The majority of residents received their genetic education in the classroom rather than through hands-on clinical experience. This could be owing to our institute's small genetics division and lack of a genetics fellowship program, both of which could help residents interact more in genetic education. However, some training programs do not have genetics divisions or genetics specialist, which may exacerbate the situation. Offering genetic electives at another school or providing training through telegenetics could be one approach.

The findings of this study add to the growing body of evidence as our PCPs in training recognize that genetic knowledge is required for practice. Harding et al. in a survey of PCPs who endorsed a responsibility to integrate genetics into their practices and expected advances in genetic medicine to expand, despite the fact that knowledge deficiency remains a problem [17]. A multidisciplinary setting can aid the integration of genetic education into different disciplines, increasing residents' exposure and attracting them to the field. We suggest a multidisciplinary clinic education that can be evaluated in large-scale, and longitudinal investigations to identify the effect on residents' genetics knowledge and future practice behavior.

In addition, our findings corroborate previously published documented knowledge gaps in the literature regarding genetic education at the PCP level. A survey of American Academy of Pediatrics members on genetic testing yielded similar results: because of the complexity 72%, of respondents refer patients to genetic services [16] (87% of the residents reported complexity of the field in our study). The sense of complexity is more likely due to a lack of knowledge about rare genetic conditions than the actual complexity. It is well-established that learning through hands-on experiences helps to connect knowledge learning to real-world patient care situations [18].

Our results were limited by a small sample size and a low response rate, which resulted in a low statistical power. Possible reasons include lack of interest to genetics, or the busy time of residents. However, following the third contact, our response rate is quite similar to the known response rate to surveys among physicians (52 percent) [19, 20]. Our findings will need to be confirmed in larger sample and in different settings, such as urban and rural training programs with a more diverse population. Future research should consider the starting sample size to accept a somewhat lower overall survey response rate in order to achieve statistical power. Funded studies with extensive questionnaires or phone interviews may give in-depth results that can aid in the development of practical techniques for introducing genetics education into residency programs so that genetics medicine can be integrated into primary care practice.

Expanding of genetic testing and genetic diseases (Fig. 1) necessitate additional evaluation of rotation structure, online tools to supplement existing educational courses, and advancement of knowledge to non-genetics health professionals. The field will not only improve access to care for patients with rare diseases but will also advance the interpretation of pharmacogenetics and personalized medicine. Our residents’ feedback could aid in the development of future questionnaires that cover topics including research experience and short rotations, among other things. None of the surveyed respondents took part in our genetic rotation, indicating that they were either unaware or uninterested in the field. One resident, for example, stated that he had no idea genetics was a recognized medical field. Increasing residents' awareness, as suggested by some residents, can be beneficial.

To our knowledge this is the first study aimed at examining genetics education perception during residency training in a large tertiary healthcare system. The study was limited by a small sample size and a low response rate, which raises concerns about the generalizability of the findings. The lower response rate could be attributed to those who did not respond ‘lack of interest’ in genetics education. We used a web-based questionnaire to collect useful information at a low cost. The use of a questionnaire requires self-reporting. Future studies should focus on examining knowledge of genetic education during residency training through testing (i.e., in-training scores) and clinical practices.

## Conclusion

Residents’ perceptions of genetic education in primary care residency programs enabled us to identify gaps in training and the potential to integrate solutions. A larger multicenter study can provide a foundation for concrete recommendations on what changes in education should occur in response to advancements in the field. This study adds to a growing body of evidence demonstrating the need for greater genetic education in residency and continuing medical education to keep physicians current on the constantly changing field of genetic testing and its application in personalized care. Additional strategies are required to increase medical students' exposure and education, as well as to remove barriers and ease primary care providers' exposure to the complexities of rare genetic diseases.

## Supplementary Information

**Additional file 1.**

## Data Availability

The datasets used and/or analysed during the current study are available from the corresponding author on reasonable request.

## Abbreviations

ACMG: The American College of Medical Genetics
CME: Continue Medical Education
PCP: Primary care provider
PGY: Post graduate year
WVU: West Virginia University
