# Supplementary material for: Genetics education in primary care residency training: satisfaction and current barriers
Source: BMC Prim Care. 2022 Jun 19;23:156. doi: 10.1186/s12875-022-01765-0 (PMC9208192; doi:10.1186/s12875-022-01765-0)
Supplement: Supplementary file 1 — Additional file 1. [file 12875_2022_1765_MOESM1_ESM.docx]

**Supplement 1.**

**Consent Form**

You are invited to participate in a web-based online survey on **“Genetic Education to Primary Care trainees.”** research. This is a research project being conducted by Dr. Nadia Falah, MD. It should take **approximately 5 minutes** to complete.

PARTICIPATION
Your participation in this survey is voluntary. You may refuse to take part in the research or exit the survey at any time without penalty. You are free to decline to answer any particular question you do not wish to answer for any reason.

BENEFITS
**The first 50 participant will receive $10 gift card as an appreciation to their participation in this research study.** Your responses may help us learn more about improving integration of genomic medicine to a primary care.

RISKS

There are no foreseeable risks involved in participating in this study.

CONFIDENTIALITY
Your survey answers will be sent to a link at Qualtrics.com where data will be stored in a password protected electronic format. Qualtrics does not collect identifying information such as your name, email address, or IP address. Therefore, your responses will remain anonymous. No one will be able to identify you or your answers. You will be asked to put your information to receive the gift card, but information will not be linked to your survey response.

CONTACT
If you have questions at any time about the study or the procedures, you may contact the research PI, Dr. Nadia Falah via phone at 304-598-4835 or via email at nadia.falah@hsc.wvu.edu

If you feel you have not been treated according to the descriptions in this form, or that your rights as a participant in research have not been honored during the course of this project, or you have any questions, concerns, or complaints that you wish to address to someone other than the investigator, you may contact the WVU Institutional Review Board at 304-293-7073 , or email IRB@mail.wvu.edu

ELECTRONIC CONSENT: Please select your choice below. You may print a copy of this consent form for your records. Clicking on the “Agree” button indicates that

- You have read the above information
- You voluntarily agree to participate
- You are 18 years of age or older
- Agree
- Disagree

**Supplement 2.**

**Questionnaire.**

Demographics

1. I am

- Pediatrics Resident
- Internal Medicine resident
- Med-Peds Resident
- Family Medicine resident
- OBGYN resident.

1. I am

- PGY1
- PGY2
- PGY3
- PGY4
- PGY5
- PGY6

1. What is your gender?

- Male
- Female
- Non-binary/Third gender
- Prefer not to say

1. What is your age?

- 25-30
- 31-35
- 36-40
- 41-45
- 46-50

1. What is your ethnicity?

- White
- Black/African
- Asian
- Middle Eastern
- Prefer not to answer

1. Which medical school did you graduate from?

- US
- Canadian
- International

1. Genetic is very important for any specialty that you can go to?

- Agree
- Disagree
- I don’t know

1. Are you considering rotating in Medical Genetics Division?

- Yes.
- No
- I don’t know
- I have already completed my rotation

1. Rank the following factors of order of importance that may motivate you to rotate in the Genetic Division?

- Personal interest
- Previous positive experience
- Influence from a mentor
- Research experience
- Future training interest
- Other [ ]

Genetics Education and Experience

1. Indicate your genetics educational experiences during your training (Choose multiple answers )
2. Grand round
3. Lectures
4. Didactic and conference sessions
5. Genetic clinic
6. Genetic rotation
7. Genetic consultation (wards)
8. Genetic research
9. Other […]
10. I have access to (Choose multiple answers)

- Medical genetics curriculum
- Block rotation
- Clinic rotation
- Genetic Lectures
- Genetic research

Genetics training satisfaction

1. Indicate the adequacy of your educational experiences in the following

| Topic | Most adequate | Adequate | Most inadequate | Inadequate |
| --- | --- | --- | --- | --- |
| Basic Genetics |  |  |  |  |
| Capturing family history |  |  |  |  |
| Initiating genetic work up |  |  |  |  |
| Basic understanding of a genetic testing report |  |  |  |  |
| Basic management surveillance to a genetic patient |  |  |  |  |
| Explaining a genetic referral to your patient |  |  |  |  |

Genetic Education Barriers

1. What are barriers in genetic education

- Complexity
- No treatment of the most genetic disease
- Boring
- Other […..]

1. How do you think we can do to best to provide genetic education?

- [……..]
